# Catabolic stress induces expression of hypoxia-inducible factor (HIF)-1α in articular chondrocytes: involvement of HIF-1α in the pathogenesis of osteoarthritis

**DOI:** 10.1186/ar1765

**Published:** 2005-05-27

**Authors:** Kazuo Yudoh, Hiroshi Nakamura, Kayo Masuko-Hongo, Tomohiro Kato, Kusuki Nishioka

**Affiliations:** 1 Department of Bioregulation, Institute of Medical Science, St. Marianna University School of Medicine, Kawasaki, Japan

## Abstract

Transcription factor hypoxia-inducible factor (HIF)-1 protein accumulates and activates the transcription of genes that are of fundamental importance for oxygen homeostasis – including genes involved in energy metabolism, angiogenesis, vasomotor control, apoptosis, proliferation, and matrix production – under hypoxic conditions. We speculated that HIF-1α may have an important role in chondrocyte viability as a cell survival factor during the progression of osteoarthritis (OA). The expression of HIF-1α mRNA in human OA cartilage samples was analyzed by real-time PCR. We analyzed whether or not the catabolic factors IL-1β and H_2_O_2 _induce the expression of HIF-1α in OA chondrocytes under normoxic and hypoxic conditions (O_2 _<6%). We investigated the levels of energy generation, cartilage matrix production, and apoptosis induction in HIF-1α-deficient chondrocytes under normoxic and hypoxic conditions. In articular cartilages from human OA patients, the expression of HIF-1α mRNA was higher in the degenerated regions than in the intact regions. Both IL-1β and H_2_O_2 _accelerated mRNA and protein levels of HIF-1α in cultured chondrocytes. Inhibitors for phosphatidylinositol 3-kinase and p38 kinase caused a significant decrease in catabolic-factor-induced HIF-1α expression. HIF-1α-deficient chondrocytes did not maintain energy generation and cartilage matrix production under both normoxic and hypoxic conditions. Also, HIF-1α-deficient chondrocytes showed an acceleration of catabolic stress-induced apoptosis *in vitro*. Our findings in human OA cartilage show that HIF-1α expression in OA cartilage is associated with the progression of articular cartilage degeneration. Catabolic-stresses, IL-1β, and oxidative stress induce the expression of HIF-1α in chondrocytes. Our results suggest an important role of stress-induced HIF-1α in the maintenance of chondrocyte viability in OA articular cartilage.

## Introduction

The breakdown or absence of oxygen homeostasis is a hallmark of many common diseases, such as cancer, myocardial infarction, and arthritis. In most normal and tumor tissues, adaptation to hypoxic conditions is critical for successful tissue expansion [1,2]. In response to down-regulation of oxygen homeostasis, cells during hypoxic challenge transiently or chronically tolerate lowered oxygen levels by means of adaptive mechanisms [1]. In mitochondrial oxidative phosphorylation, oxygen is the terminal electron acceptor during ATP production. Several enzymatic reactions require oxygen as a substrate [3,4]. Responses to hypoxia include a metabolic shift to anaerobic glycolysis as well as the initiation of neoangiogenesis via the expression of angiogenic factors to increase the opportunity for oxygen to reach the tissue [1-5]. Oxygen homeostasis and its down-regulation are involved in the pathogenesis of common diseases [3].

It is well known that the transcription factor hypoxia-inducible factor 1 (HIF-1) appears to be one of the major regulators of the hypoxic response [3,6]. HIF-1 controls hypoxic expression of erythropoietin, as well as the expression of genes with metabolic functions such as glucose transport and metabolism, and angiogenic factors such as vascular endothelial cell growth factor (VEGF) [6-8]. HIF-1 is a heterodimer of the PAS subfamily of basic-helix-loop-helix transcription factors, and it consists of the subunit HIF-1α (120 kDa), produced in response to hypoxia, and the constitutively expressed HIF-1α (91 to 94 kDa) subunit [9]. HIF-1 protein accumulates and activates the transcription of genes that are of fundamental importance for oxygen homeostasis, including genes involved in energy metabolism, angiogenesis, vasomotor control, apoptosis, proliferation and matrix production, under hypoxic conditions [6,8,9].

Articular cartilage is an avascular tissue lacking a capillary network, in which oxygen is limited due to its delivery via diffusion through the synovial fluid. It is well known that there is a physiological gradient of oxygenation within articular cartilage [10-12]. It has been reported that the partial pressure of O_2 _in synovial fluid in joints affected by osteoarthritis (OA) is between 40 and 85 mmHg, corresponding to an oxygen concentration of approximately 6 to 11% [13]. Since O_2 _must enter from the cartilage surface, the concentration of oxygen is approximately 6% at the surface zone of the articular tissue and less than 1% in the deep zone. We histologically examined the oxygen gradation in articular cartilage tissue by immunofluorescence staining with a specific probe. We performed the analysis in human articular cartilage tissue in patients undergoing arthroplastic knee surgery. The levels of immunostaining revealed an O_2 _tension (approximately 3 to 8%) at the surface of the cartilage similar to that in positive control tumor tissues with already known O_2 _tension. There is a general consensus that articular chondrocytes are adapted to hypoxic conditions. Since HIF-1α expression is associated with low O_2_, this factor may play a role in chondrocyte survival and the maintenance of fundamental homeostasis in the normally hypoxic articular cartilage. In addition, degeneration of articular cartilage may directly influence the chondrocyte microenvironment, especially cellular adaptation to hypoxic conditions, in articular cartilage. Even a slight change may affect the adaptative hypoxic conditions of chondrocytes, resulting in alteration of the cellular microenvironment that is involved in the maintenance of articular cartilage. Indeed, more recently it has been demonstrated that HIF-1α is expressed in OA articular cartilage [14]. However, the exact role of this factor in the pathogenesis of OA remains unclear.

We postulated that HIF-1α could play an important role as a survival factor protecting tissue against catabolic changes during the progression of OA. Our data show here for the first time a correlation between the levels of expression of HIF-1α and degeneration of articular cartilage in patients with OA. To clarify the role of HIF-1α in the pathogenesis of OA, we investigated whether or not hypoxia and catabolic factors (IL-1β and H_2_O_2_) affected the expression of HIF-1α, energy generation, cartilage matrix production, and apoptosis in OA chondrocytes.

We also report evidence for the action of HIF-1α as a chondrocyte survival factor in OA.

## Materials and methods

### Preparation of human articular cartilage samples

Donor OA cartilage samples were obtained from knee joints of OA patients undergoing arthroplastic knee surgery (seven OA patients) after obtaining the patients' informed consent. The characteristics of patients are summarized in Table 1. Each sample was cut and divided into two pieces: one was used for histological evaluation and the other was stored at -30°C for later analysis by real-time PCR analysis.

Each cartilage sample was evaluated histologically and macroscopically for the degree of degeneration according to the scales of Mankin and colleagues and of Collins [15,16]. Articular cartilage samples with subchondral bones were fixed for 2 days in 4% paraformaldehyde solution and then decalcified in 4% paraformaldehyde containing 0.85% sodium chloride and 10% acetic acid. Tissues were dehydrated in a series of ethanol solutions and infiltrated with xylene and before being embedded in paraffin and cut into 6-μm sections. Sections were deparaffinized through sequential immersion in xylene and a graded series of ethanol solutions in accordance with conventional procedures. Sections were also stained with safranin O-fast green to determine the loss of proteoglycans [17].

### Chondrocyte isolation and culture

Human articular cartilage samples were obtained from knee joints during arthroplastic surgery for OA (*n *= 7, one male, six females, 61, 62, 64, 66, 67, 68, 72 years old) after obtaining the patients' informed consent. Cartilage tissues were cut into small pieces, washed in PBS, and digested in Dulbecco's modified Eagle's medium (DMEM; Sigma, St. Louis, MO) containing 1.5 mg/ml collagenase B (Sigma). Digestion was carried out at 37°C overnight on a shaking platform. Cells were centrifuged, washed with PBS, and plated with fresh DMEM. Chondrocytes were cultured in DMEM supplemented with 10% heat-inactivated fetal calf serum, 2 mM L-glutamine, 25 mM HEPES (2-[4-(2-hydroxyethyl)-1-piperazinyl] ethanesulfonic acid), and 100 units/ml penicillin and streptomycin at 37°C in a humidified 5% CO_2 _atmosphere [18].

### Chondrocyte culture under hypoxic conditions

Human chondrocytes were dispensed into a 10-cm culture plate. The plates were placed in a sealed hypoxia chamber (Billups-Rothenberg, Del Mar, CA, USA) equilibrated with a humidified 5% CO_2 _atmosphere or with certified gas containing 1% O_2_, 5% CO_2_, and 94% N_2 _[19,20]. In this hypoxia chamber system, approximately 5 to 6% O_2 _tension was observed after 15 min of gas flow (20 l/min). The O_2 _tension in the culture medium was monitored with an oxygen meter (Fuso Rekaseihin Ltd, Tokyo, Japan) as described by the manufacturer. We monitored the O_2 _tension with an oxygen meter to maintain the concentration of approximately 6%. When so indicated, recombinant human IL-1β (10 ng/ml; Sigma) or H_2_O_2 _(10.0 μM; Wako Pure Industries, Tokyo, Japan) was added, and the cells were incubated under normoxic or hypoxic culture conditions at 37°C. As a positive control, COCl_2 _(150 μM; Sigma), a chemical inducer of HIF-1, was added to the cells during the incubation time in normoxia or hypoxia [20].

In other experiments, human chondrocytes were cultured in the presence or absence of a phosphatidylinositol 3-kinase (PI3K) inhibitor LY294002 (Sigma), a p38 mitogen-activated protein kinase (MAPK) inhibitor SB203580 (Sigma), and extracellular signal-regulated kinase (ERK 1/2) inhibitor PD98059 (Wako).

### Immunoblotting

Cells were lysed in boiling sample buffer as suggested by the manufacturer (Sigma). Samples were then homogenized by repeated aspiration through a 26-gauge needle. Cellular proteins were resolved by SDS-PAGE (12.5% acrylamide) and were transferred to nitrocellulose membranes. Blots were incubated for 2 hours in Tris-buffered saline/Tween 20 (TBST; 10 mM Tris/HCL, pH 8.0, 150 mM NaCl, and 0.2% Tween 20) containing 2% powdered skimmed milk and 1% bovine serum albumin. After three washes with TBST, membranes were incubated for 2 hours with the primary antibody to HIF-1α (diluted 1000-fold in TBST) (Santa Cruz Biotechnology Inc, Santa Cruz, CA) and for 1 hour with horseradish-peroxidase-conjugated goat antimouse IgG (diluted 1000-fold) (DAKO, Glostrup, Denmark). Bound antibodies were detected using an ECL detection kit (Amersham Bioscience KK, Tokyo, Japan). Densitometry of the signal bands was analyzed with Image Gauge version 4.0 (FUJI Photo Film, Tokyo, Japan).

### Proteoglycan production in chondrocytes

Chondrocyte activity was measured by the production of glycosaminoglycan (GAG) from cultured chondrocytes. Chondrocytes were cultured under either normoxic or hypoxic conditions using the sealed hypoxia chamber. After 24 hours of incubation, we collected the cells and supernatant. The amount of GAG in the supernatant was measured by using a spectrophotometric assay with dimethylmethylene blue (Aldrich Chemical, Milwaukee, WI, USA) measured at 540 nm using shark chondroitin sulfate (Sigma) as a standard [19].

### Measurement of lactic acid in cultured chondrocytes

Supernatants from chondrocyte cultures were collected after 24 hours under normoxic or hypoxic conditions. Lactic acid was determined by a colorimetric assay (Sigma) at 540 nm in accordance with the manufacturer's instructions. Lactic acid levels were normalized to total protein content as measured by the Bradford assay (Bio-Rad, Hercules, CA, USA) [21].

### ATP levels in cultured chondrocytes

Chondrocytes were collected after a 24-hour incubation under normoxic or hypoxic conditions. The ATP Bioluminescence assay kit CLS II (Roche, Heidelberg, Germany) was used. The assay is based on the light-emitting oxidation of luciferin by luciferase in the presence of extremely low levels of ATP. After collecting the chondrocytes by scraping, cells were centrifuged for 10 min at 500 × **g **in the cold. Chondrocytes pellets were extracted by boiling 100 mM Tris (tris(hydroxymethyl)aminomethane) buffer containing 4 mM EDTA (ethylenediaminetetraacetic acid) for 2 min in order to inactivate NTPases. Cell remnants were removed by centrifugation at 1000 × **g**. Supernatants were removed and placed on ice. Determination of free ATP was as outlined in the manufacturer's protocol. Light emission was measured at 562 nm using a luminometer. ATP levels were normalized to protein content as measured by the Bradford assay (Bio-Rad) [19].

### RT-PCR

Total RNA was extracted from articular cartilage by acid guanidine–phenol–chloroform extraction using ISOGEN ^® ^(Nippon Gene Inc, Tokyo, Japan). First-strand complementary DNA (cDNA) was synthesized with Superscript II reverse transcriptase. PCR amplification was performed using specific primers (Table 2). The PCR products were analysed by electrophoresis in 2% agarose gels stained with ethidium bromide, and bands were visualized and photographed under ultraviolet excitation.

### Real-time PCR

For PCR analyses, cDNA from triplicate dishes from four independent experiments (24 hours of hypoxia or normoxia) were diluted to a final concentration of 10 ng/ μl. Quantitative real-time RT-PCR was performed with a TaqMan Universal Mastermix (Biosystems Inc, Foster City, CA). cDNA (50 ng) was used as template to determine the relative amounts of mRNA by real-time PCR (ABI 7700 sequence detection system) using specific primers and probes (Table 2). The reaction was conducted as follows: 95°C for 4 min, and 40 cycles of 15s at 95°C and 1 min at 60°C (21). To standardize mRNA levels, we amplified 18S rRNA as an internal control and calculated using Microsoft Excel.

### Antisense oligonucleotide treatment of chodrocytes

HIF-1α depletion in chondrocytes was accomplished by using antisense oligonucleotide (ODN) loading using phosphorothioate derivatives of antisense (5'-GCCGGCGCCCTCCAT-3') or control sense (5'-ATGGAGGGCGCCGGC-3') oligonucleotides. Antisense HIF-1α ODN and control ODN were designed and synthesized by BIOGNOSTIK (Göttingen, Germany). Scrambled oligonucleotide was used as control. Chondrocytes were washed in serum-free medium and then in medium containing 20 mg/ml transfection reagent (Qiagen Inc, Valencia, CA, USA) with 2 μM HIF-1α antisense or control ODN. Cells were incubated for 4 hours at 37°C and then replaced with medium containing growth factors. The cellular uptake efficiency was monitored by fluorescein-isothiocyanate-labeled ODN (transfection efficiency approximately 60 to 70% after 4 hours of treatment). The transfection efficiency detected by fluorescein-isothiocyanate-labeled ODN was maintained after a further 24 hours of incubation. Treated cells were cultured in hypoxic or normoxic conditions for the indicated periods of time (24 hours) in each experiment. HIF-1α mRNA was quantified by RT-PCR and western blotting analysis as described above. Data were analyzed for four independent experiments.

### Apoptosis

Human subconfluent chondrocytes were cultured in the presence of 10 ng/ml IL-1β for 24 hours under the normoxic or hypoxic conditions described above. Cellular apoptosis was detected using the Apoptosis detection kit (TdT *in situ *apoptosis detection kit: R&D systems Inc., MN, USA) in chondrocyte cell cultures in accordance with the manufacturer's protocol. The kit was used to identify apoptotic cells by detecting DNA fragmentation through a combination of enzymology and immunohistochemistry techniques. Biotinylated nucleotides are incorporated into the 3'-OH ends of the DNA fragments by terminal deoxynucleotidyl transferase (TdT). Cells containing fragmented nuclear chromatin characteristic of apoptosis exhibit a brown nuclear staining. Apoptosis was assessed by measuring the percentage of apoptotic nuclei in each sample [22,23].

### Statistical analysis

Results were expressed as means ± standard deviations. Data were analyzed by a nonparametric statistical analysis. An analysis resulting in value of *P *< 0.05 was considered statistically significant.

## Results

### HIF-1α mRNA expression in articular cartilage from patients with OA

**Figure 1:**
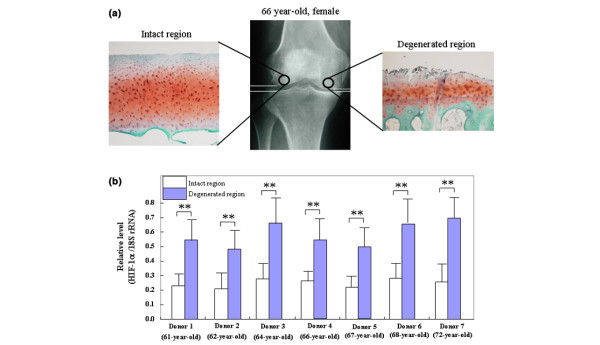
Levels of HIF-1α mRNA in the articular cartilage from patients with osteoarthritis (OA). **(a)**Representative x-ray film of knee joint and safranin-O staining for hypoxia-inducible factor 1α(HIF-1α) in the degenerated region and intact region of articular cartilage from a 66-year-old woman with OA. Original magnification of histological sections × 200. **(b) **The mRNA levels of HIF-1α were higher in the degenerated regions than in the intact regions from the same OA sample.

To clarify the expression of HIF-1α mRNA in human OA cartilage, the real-time PCR analysis for HIF-1α was performed with donor-matched pairs of intact and degenerated articular cartilage isolated from the same OA sample. Fig. 1a shows a representative safranin-O staining in the degenerated region and intact region of articular cartilage from OA patients. The levels of HIF-1α mRNA in all seven donor articular cartilage samples were higher in the degenerated regions than in the intact regions (Fig. 1b).

### Catabolic factors induce the expression of HIF-1α in human articular cartilage

**Figure 2:**
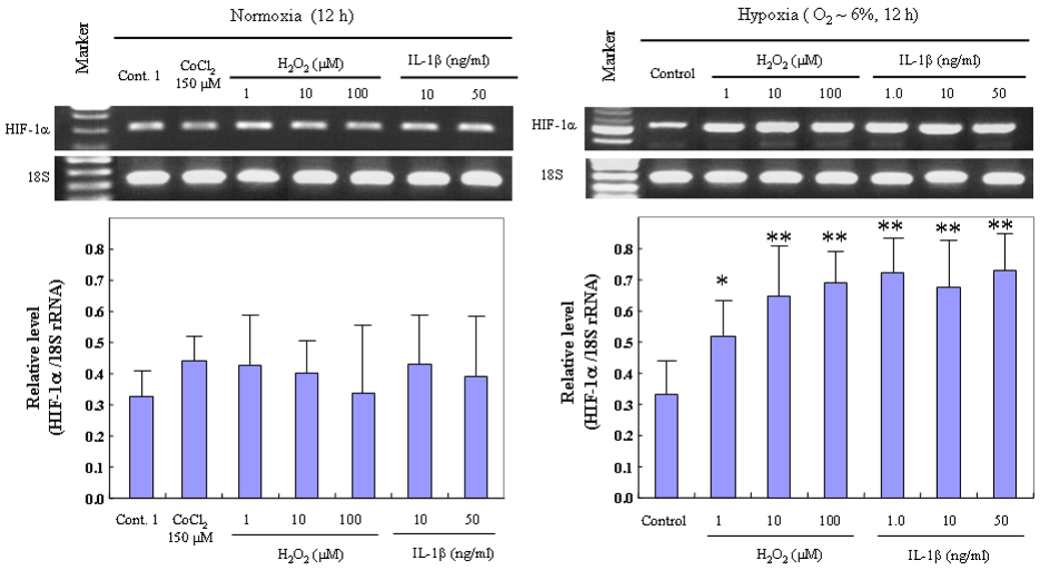
IL-1β and H_2_O_2 _induce the expression of HIF-1α mRNA in human articular chondrocytes. Under normoxic culture conditions, mRNA levels of hypoxia-inducible factor 1α (HIF-1α) were observed in cultured chondrocytes, whereas HIF-1α protein was undetected in the cells. HIF-1α mRNA was accelerated by IL-1β or H_2_O_2 _in cultured chondrocytes under hypoxic conditions. Cobalt chloride (CoCl_2_), chemical inducer of HIF-1, was used for the positive control. *P < 0.05, **P < 0.01 compared with the control. Cont., control.

**Figure 3:**
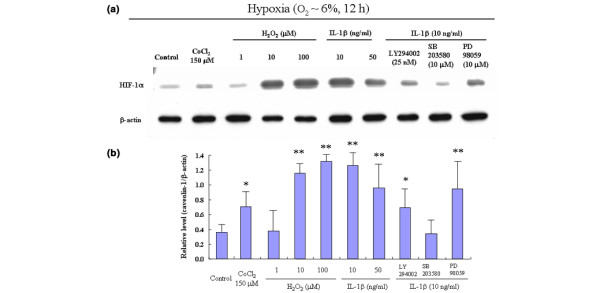
Catabolic factors induce the expression of HIF-1α protein in human articular cartilage. **(a)**Hypoxia-inducible factor 1α (HIF-1α) protein was accelerated by IL-1β or H_2_O_2_in cultured chondrocytes under hypoxic conditions. **(b)**Under hypoxic conditions, the inhibitors of PI3K and p38 mitogen-activated protein kinase (MAPK) reduced protein levels of IL-1β-induced HIF-1α expression. Cobalt chloride (CoCl_2_), chemical inducer of HIF-1, was used for the positive control. *P < 0.05, **P < 0.01 compared with the control. LY294002: phosphatidylinositol 3-kinase inhibitor; SB203580: p38 mitogen-activated protein kinase inhibitor; PD98059: extracellular signal-regulated kinase inhibitor.

To clarify the effect of catabolic factors on HIF-1α expression in human articular cartilage, the quantitative real-time PCR and western blotting analysis were performed under normoxic and hypoxic culture conditions. In normoxic culture conditions, mRNA levels of HIF-1α were observed in cultured chondrocytes, whereas HIF-1α protein was undetected regardless of stimulation of IL-1β and H_2_O_2 _(Fig. 2). Under hypoxic culture conditions, both HIF-1α mRNA and protein were detected in cultured chondrocytes (Figs 2, 3). The expression of HIF-1α was significantly accelerated by the chondrocyte catabolic factors IL-1β and H_2_O_2 _(Figs 2, 3). Under hypoxic conditions, the inhibitors of PI3K and p38 kinase caused a significant decrease in the catabolic-factor-induced HIF-1α expression (Fig. 3a, b). Data from four independent experiments were analyzed.

### Role of HIF-1α in free ATP production in human articular chondrocytes

**Figure 4:**
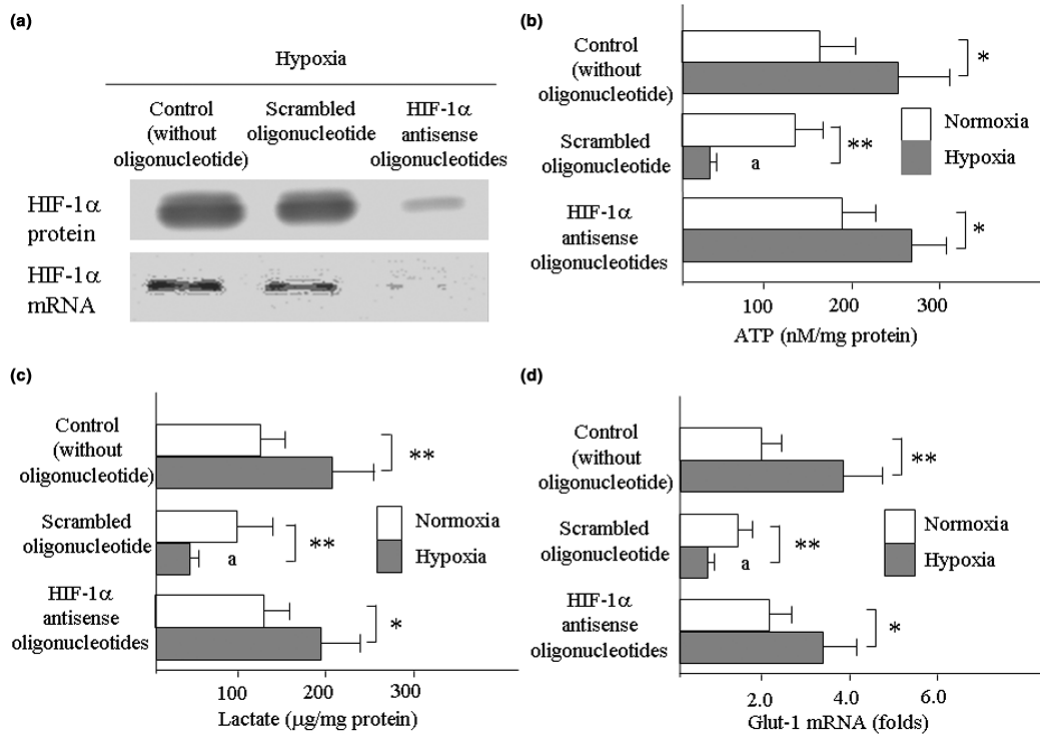
Effect of HIF-1α on ATP production and glycolysis in human articular cartilage. **(a)**Hypoxia-inducible factor 1α (HIF-1α) depletion by antisense oligonucleotide was assessed by RT-PCR and western blotting analyses. HIF-1α mRNA and protein expressions were reduced in antisense HIF-1α-treated chondrocyte populations. Scrambled oligonucleotide was used as control oligonucleotide. Representative data from four independent experiments are shown. **(b)**In hypoxia, HIF-1α-deficient chondrocytes showed a significant decrease of free ATP in comparison with control oligonucleotide-treated chondrocytes. Statistical differences were calculated using data from four independent experiments. **(c, d) **The levels of lactate (c) and glucose transporter-1 (Glu-1) (d) were increased in the scrambled ODN-treated chondrocytes under hypoxic culture conditions compared with normoxic culture condition. In HIF-1α-deficient chondrocytes, both glycolytic activities were reduced under hypoxic conditions. Statistical differences were calculated using data from four independent experiments. ^a^*P *< 0.01, control oligonucleotide hypoxia vs HIF-1α-deficient hypoxia; *P < 0.05, **P < 0.01.

To study the role of HIF-1α in chondrocyte energy production, we measured the free ATP levels of cultured chondrocytes under normoxic and hypoxic culture conditions. In control chondrocytes, the levels of free ATP in hypoxia were significantly higher than in normoxia. Under hypoxic conditions, HIF-1α-deficient chondrocytes showed a significant decrease of free ATP in comparison with control ODN-treated chondrocytes (Fig. 4b). In HIF-1α-deficient chondrocytes, free ATP production was approximately 20% of control cells under hypoxic conditions. In contrast, although HIF-1α-deficient chondrocytes showed a slight decrease of energy generation under normoxic conditions, there was no statistically significant difference in energy generation between the three groups (normal chondrocytes, antisense ODN-treated chondrocytes, and chondrocytes treated with the scrambled ODN). Data for four independent experiments were analyzed.

### Influence of HIF-1α deficiency on glycolysis in human chondrocytes

As shown in Fig. 4c, d, significant increases of lactic acid (c) and glucose transporter-1 (d) were observed in control ODN-treated chondrocytes under hypoxic culture conditions compared with normoxic culture conditions. In contrast, HIF-1α-deficient chondrocytes showed a complete loss of the induced increases in glycolytic activities even under hypoxic culture conditions.

Under normoxic conditions, HIF-1α-deficient chondrocytes showed a slight decrease of energy generation; however, there was no statistically significant difference in energy generation between control chondrocytes and antisense HIF-1α-treated chondrocytes. Data for four independent experiments were analyzed.

### Proteoglycan production from chondrocytes in different oxygen tension

**Figure 5:**
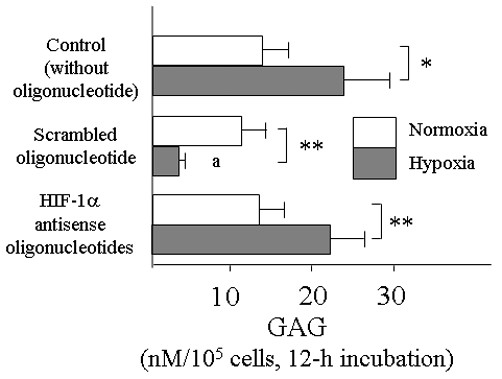
Glycosaminoglycan production and apoptosis induction in HIF-1α-deficient chondrocytes. In the scrambled oligonucleotide-treated groups, the amount of glycosaminoglycan (GAG) produced by cultured chondrocytes was higher under hypoxic conditions than under normoxic conditions. Under hypoxic conditions, GAG levels decreased in chondrocytes deficient in hypoxia-inducible factor 1α (HIF-1α). Statistical differences were calculated using data from four independent experiments. ^a^*P *< 0.01, control oligonucleotide hypoxia vs HIF-1α-antisense hypoxia; *P < 0.05; **P < 0.01.

To test whether HIF-1α-mediated alteration affects the potential to produce matrix proteins in chondrocytes, we determined the amount of GAG produced by cultured chondrocytes. We observed large increases in the concentration of GAG in control ODN-treated cultures under hypoxia compared with normoxia. GAG levels were decreased in HIF-1α-deficient chondrocytes under hypoxia to approximately 35% of control levels (Fig. 5). Data for four independent experiments were analyzed.

### Apoptosis induction by catabolic factors in HIF-1α-deficient chondrocytes

**Figure 6:**
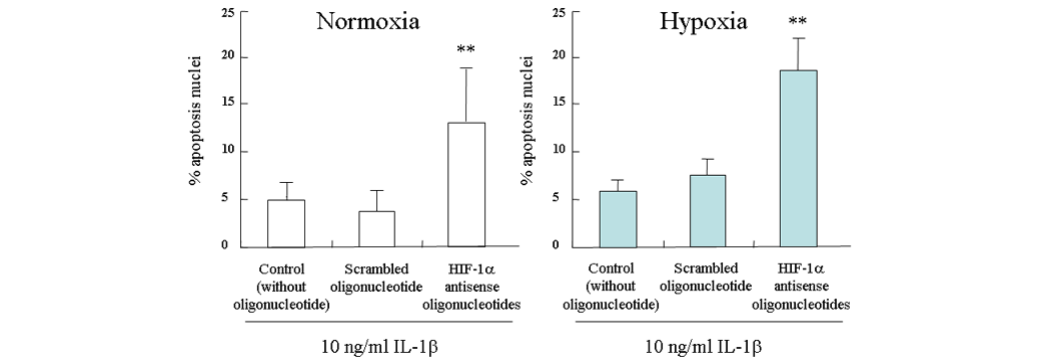
Apoptosis induction in HIF-1α-deficient chondrocytes. IL-1β was used for apoptosis induction under hypoxic or normoxic conditions in chondrocytes lacking hypoxia-inducible factor 1α (HIF-1α), treated with oligonucleotide, and cultured without oligonucleotide, and also not exposed to the oligonucleotide or HIF-1α antisense nucleotides. IL-1β-induced apoptosis was significantly increased in HIF-1α-deficient chondrocytes compared with chondrocytes treated with scrambled oligonucleotide under both normoxic and hypoxic culture conditions. Statistical differences were calculated using data from four independent experiments. **P < 0.01, control oligonucleotide group vs HIF-1α-antisense group.

Under hypoxic conditions, IL-1β-induced apoptosis was increased in hypoxic chondrocytes lacking HIF-1α, to twice that of control ODN-treated chondrocytes (Fig. 6). Even under normoxic conditions, HIF-1α-deficient chondrocytes showed significantly increased levels of apoptosis compared with their control counterparts. Data for four independent experiments were analyzed.

## Discussion

Our findings show the potential involvement of HIF-1α expression in the progression of articular cartilage degeneration. In patients with OA, stronger expression of HIF-1α mRNA in chondrocytes was observed in degenerating regions than in intact regions from the same articular cartilage samples. Our findings in human articular cartilage tissues indicate for the first time that expression of HIF-1α mRNA is closely involved in the progression of articular cartilage degeneration.

The HIF-1 complex is ubiquitous, and the presence of this complex in growth-plate chondrocytes has been documented recently [24-26]. Schipani and colleagues reported that in HIF-1α-null mice, hypoxic chondrocytes showed massive cell death in cartilaginous elements such as the chondrosternal junction of the ribs and growth plate, suggesting that HIF-1α is not only crucial for survival of hypoxic chondrocytes, but also modulates the process of chondrocyte proliferation, differentiation, and growth arrest in growth-plate chondrocytes [26]. More recently, Coimbra and colleagues also showed that HIF-1α is expressed in cultured cartilage and chondrocytes under both normoxic and hypoxic conditions [14]. Their findings of HIF-1α expression in chondrocytes are basically consistent with our results from both human and rat OA cartilages. However, from their data, it remained unclear whether HIF-1α expression in chondrocytes is related to the degeneration of articular cartilage *in vivo*. Indeed, Coimbra and colleagues showed that HIF-1α was expressed not only in normal chondrocytes and cartilage but also in OA chondrocytes, under both hypoxic and normoxic conditions *in vitro*. In our present study, HIF-1α protein was undetected in chondrocytes under normoxic conditions. It is well known that cellular HIF-1α is not detected in normoxia [27-29]. Under normoxic conditions, the HIF-1α protein undergoes ubiquitination and rapid degeneration in proteasomes [30].

Our data suggest that chondrocyte catabolic factors IL-1β and oxidative stress (oxidative free radicals) may induce the expression of HIF-1α in articular chondrocytes. IL-1 has been shown both to inhibit chondrocyte anabolic activity, including the down-regulation of proteoglycan synthesis, and to stimulate catabolic activity, including production of metalloproteinases [31,32]. IL-1 also stimulates chondrocyte expression of inducible nitric oxide synthesis, iNOS, which results in an increase in NO production [33]. Numerous reports have already demonstrated that oxidative stress acts as a catabolic factor in articular cartilage [34-38]. Articular chondrocytes actively produce endogenous reactive oxygen species, O_2_^- ^[35], NO [36], ^-^HO [37], and H_2_O_2 _[3]). Oxidative damage in cartilage may affect chondrocyte function, resulting in changes in cartilage homeostasis that are relevant to cartilage aging and the development of OA. Our data indicated that in cultured chondrocytes, both mRNA and protein levels of HIF-1α were up-regulated by both IL-1β and H_2_O_2 _under hypoxic but not normoxic conditions. These findings suggest that OA-related catabolic stresses (IL-1β, H_2_O_2_) induce the expression of HIF-1α in the degenerated articular cartilages as degeneration progresses.

Interestingly, besides hypoxia, many cytokines and growth factors have been shown to be capable of stabilizing and activating HIF-1α under normoxic conditions. Stimulation of cultured synovial fibroblasts with IL-1β and TNFα increases levels of HIF-1α mRNA. Moreover, incubation with IL-1β leads to stabilization of HIF [39]. Our results of catabolic stress-induced expression of HIF-1α in chondrocytes are consistent with these findings. These findings suggest that HIF-1α may, at least in part, have some role in the pathogenesis of inflammatory arthritis even under normoxic conditions, although further studies are needed to clarify this issue. Also, these findings, including our results, provide evidence to support the idea that OA-related catabolic factors (IL-1β etc.) induce HIF-1α during the progression of cartilage degeneration.

In this context, we also studied the signal transduction pathways involved in stress-induced HIF-1α expression in chondrocytes. It has been reported that p38 MAPK, PI3K, and ERK MAPK pathways are responsible for the stress-induced responses in a variety of cells [40-42]. We found that both IL-1β and H_2_O_2 _induced a prolonged activation of p38 in chondrocytes (data not shown). Under hypoxic conditions, the inhibitors of PI3K and p38 kinase caused a significant decrease in catabolic-factor-induced HIF-1α expression; this finding supports the idea that PI3K and p38 kinase, but not ERK, activation are required for catabolic stress-induced HIF-1α expression in chondrocytes in hypoxia. Local accumulation of a regulating protein to adapt to hypoxia may be mediated, at least in part, by p38 and PI3K in articular chondrocytes. In addition, we have focused on the redox factor 1 (Ref-1, also known as APE, HAP1, and APEX), a ubiquitous multifactorial protein that is a redox-sensitive regulator of mutifactorial transcription factors, including nuclear factor κB, c-myc gene, activating protein-1, and HIF-1α. Ref-1 may play a critical role in the regulation of endothelial cell fate in response to pathophysiological stimuli such as hypoxia [43]. We have studied the interactions between Ref-1 and HIF-1 activity in OA chondrocytes (data not shown).

Our *in vitro *data clearly indicate that expression of HIF-1α is responsible for the energy generation and cellular survival of hypoxic chondrocytes. We have shown that HIF-1α activity is essential for regulation of glycolysis, energy generation, synthesis of cartilage matrix proteins, and cell survival in OA chondrocytes under hypoxic conditions. HIF-1α-null chondrocytes did not maintain their viability; energy generation, and matrix production under normoxic and hypoxic conditions. In addition, HIF-1α-null chondrocytes showed accelerated apoptosis induction by IL-1β, suggesting that HIF-1α has an important role in the survival of tissues that lack a functional vasculature, such as articular cartilage.

Articular cartilage adapts to hypoxic conditions, since the cartilage is an avascular tissue. Nutrition and oxygen for articular cartilage are supplied from the synovial fluid. Even a surface zone of articular cartilage has lower oxygen tension (approximately 6%) than synovial fluid (approximately 6 to 15%) [10-13]. There is an oxidative gradient in articular cartilage. Oxygen homeostasis in normal articular cartilage is maintained under hypoxic conditions. During the progression of cartilage degeneration, OA-related catabolic stresses, mechanical and chemical, including IL-1β and oxidative stress, could induce the degradation of the extracellular matrix and decrease chondrocyte viability, resulting in the down-regulation of chondrocyte environment and the further degeneration of articular cartilage. The OA-related changes may also affect oxygen tension and the hypoxic conditions in articular cartilage. Breakdown of oxygen homeostasis in articular cartilage may influence the chondrocytes adapted to hypoxic conditions within the articular cartilage. Although further studies are needed to clarify the exact mechanism of HIF-1α expression, HIF-1α may be expressed in response to change of cellular microenvironment, especially O_2 _tension, in the tissue. Jewell and colleagues reported that HIF-1α was up-regulated by reoxygenation [44]. Their findings suggest that expression of HIF-1α may be influenced by the cellular microenvironment. When there is deviation from the stable condition in terms of O_2 _tension, HIF-1α expression may be influenced to maintain the cellular homeostasis. Articular chondrocytes that are well adapted to hypoxia into the tissue may express HIF-1α with the deviating from adaptation to hypoxia. We postulated that HIF-1α is expressed in response to catabolic change in articular cartilage to maintain the cell viability and readaptation to hypoxia. Catabolic stress during the development of OA could influence the chondrocyte adaptation to hypoxia in the tissue.

Our findings of catabolic-factor-induced HIF-1α expression in chondrocytes provide evidence to support the idea that HIF-1α expression is up-regulated in response to catabolic degeneration of articular cartilage. Although genes under the control of HIF-1α have not yet been analyzed in OA, stress-induced HIF-1α may lead to the expression of anti-apoptotic factors or act as a chondroprotective factor to maintain chondrocyte viability in the face of catabolic changes in articular cartilage. Many molecular aspects of HIF-1 signaling should be also studied to further clarify the role of HIF-1 in the pathogenesis of OA.

## Conclusion

Taken together, our findings show for the first time that exposure to IL-1β and oxidative stress induce HIF-1α expression in degenerated articular cartilage, which is mediated, at least in part, by activation of PI3K and p38 kinase. Our data suggest a potential for HIF-1α in the maintenance of chondrocyte viability in articular cartilage challenged by progressive articular cartilage degeneration, and they provide new insights into pathogenesis of OA.

## Abbreviations

DMEM = Dulbecco's modified Eagle's medium; ERK = extracellular signal-regulated kinase; GAG = glycosaminoglycan; HIF-1α = hypoxia-inducible factor 1α; IL-1 = interleukin-1; MAPK = mitogen-activated protein kinase; MCL = medial collateral ligament; OA = osteoarthritis; ODN = oligonucleotide; PBS = phosphate-buffered saline; PI3K = phosphatidylinositol 3-kinase; TBST = Tris-buffered saline/Tween 20; TdT = terminal deoxynucleotidyl transferase; Tris = tris(hydroxymethyl)aminomethane.

## Competing interests

The authors declare that they have no competing interests.

## Authors' contributions

KY carried out *in vitro *studies (cell culture), participated in the design of the study, conducted sequence alignment, and drafted the manuscript. HN, K H-M, TK, and KN conceived the study, participated in its design and coordination, and helped to draft the manuscript. All authors read and approved the final manuscript.

## Figures and Tables

**Table 1:** Characteristics of patients with osteoarthritis

				Mankin grade	
Donor	Age (y)	Sex	Disease duration (y)	Intact region	Degenerated region

1	61	Female	5.5	3	7
2	62	Female	6.0	4	9
3	64	Male	8.4	3	8
4	66	Female	11.5	2	9
5	67	Female	9.6	2	9
6	68	Female	8.4	2	8
7	72	Female	9.0	3	9

**Table 2:** Sequences of PCR primers and probes

	Primer (5'-3')	Probe
c-Jun	fw: TGCATGCTATCATTGGCTCATAC	CCCGGCAACACACA-MGB
	rv: CACACCATCTTCTGGTGTACAGTCT	
HIF-1α	fw:CTATGGAGGCCAGAAGAGGGTAT	AGATCCCTTGAAGCTAG-MGB
	rv:CCCACATCAGGTGGCTCATAA	
Glucose transporter-1	fw:GGGCATGTGCTTCCAGTATGT	CAACTGTGCGGCCCCTACGTCTTC
	rv:ACGAGGAGCACCGTGAAGAT	

fw, forward; rv, reverse
